# Knowledge and Perceptions of Self-Medication Among Bachelor of Medicine and Bachelor of Surgery (MBBS) Students: A Cross-Sectional, Questionnaire-Based Study

**DOI:** 10.7759/cureus.65269

**Published:** 2024-07-24

**Authors:** Abhijay Mehta, Ravinder K Gupta, Anirudh Mahajan, Anisha Kapoor, Damanpreet Singh

**Affiliations:** 1 Paediatrics, Acharya Shri Chander College of Medical Sciences and Hospital, Jammu, IND

**Keywords:** self-medication, mbbs, medical students, perception, knowledge

## Abstract

Introduction: Self-medication (SM) is a common practice worldwide, and the irrational use of drugs is a cause of concern. Self-medication has various forms, including taking medications without a physician’s prescription, using a previous prescription for a similar condition, or using drugs obtainable at home without getting a physician’s advice. The youth are highly influenced by the media and the internet, which promote SM behavior.

Aims and objectives: To determine the knowledge and perceptions of SM in Bachelor of Medicine and Bachelor of Surgery (MBBS) students in all professional years, including interns in Acharya Shri Chander College of Medical Sciences (ASCOMS) and Hospital, Jammu.

Material and methods: The present study was conducted at ASCOMS Hospital, Jammu. A total of 200 students of MBBS were included in the study. A self-structured questionnaire was used to assess the knowledge and awareness level. The collected data were recorded in a Microsoft Excel sheet (Microsoft Corp., Redmond, WA) and analyzed with the help of IBM SPSS Statistics software for Windows, version 21.0 (IBM Corp., Armonk, NY).

Result: The present study reveals that 60% of the subjects were males and 65% of the students practiced SM, which indicates that SM is highly prevalent amongst MBBS students. It was observed that minor illness and quick relief were the major reasons for SM. Further, headache, cold/cough, and fever were the major indications for SM, whereas antipyretics and analgesics were the most commonly used drugs for SM.

Conclusion: The present study concluded that there was a high prevalence of SM practice among medical students, especially among females. However, the knowledge and perceptions of SM were not insufficient.

## Introduction

Around the world, self-medication (SM) is widespread, and excessive drug use is concerning. "Use of over-the-counter medication (OTC) to treat self-diagnosed symptoms or disorders, or for the continuation and reuse of prescribed medications for recurrent diseases" is how the World Health Organization (WHO) defines SM [1].

Self-medication has various forms, including taking medications without a physician’s prescription, using a previous prescription for a similar condition, or using drugs obtainable at home without getting a physician’s advice.

Self-medication may occur for a variety of reasons, including insufficient time to see a doctor, incompetence to make an urgent appointment, relative remoteness from surrounding hospitals and clinics, a lack of slots for prompt treatment from a government hospital during busy hours, and exorbitant consultation fees. [2]. 

Self-medication has the potential to be a severe problem that can result in a few issues, including adverse drug reactions, an increase in antibiotic resistance, and a waste of resources [3].

Additional potential hazards linked to SM encompass misdiagnosing oneself, concealing a serious underlying illness, and neglecting to swiftly seek medical assistance afterward. More serious hazards include the possibility of drug dependence or misuse, errors in dosage, storage, administration technique, and medication selection, as well as the inability to identify contraindications and possible combinations with food or other medications.

The youth are highly influenced by the media and the internet, which promote SM behavior [4]. The increased advertising of pharmaceuticals poses a larger threat of SM to the younger population in general. This raises concerns about incorrect self-diagnosis, drug interaction, and use of drugs other than for the original indication [5].

Given the above, the present study was conducted to assess the knowledge and perception regarding SM among the Bachelor of Medicine and Bachelor of Surgery (MBBS) students of Acharya Shri Chander College of Medical Sciences (ASCOMS) and Hospital, Jammu, India.

This study aimed to determine the knowledge and perceptions regarding SM among MBBS students in all professional years, including interns in ASCOMS Hospital, Jammu.

## Materials and methods

The current cross-sectional study was conducted to investigate the knowledge and perception of self-medication among MBBS students (including interns) at ASCCMS Hospital, Jammu, India. The study was conducted over five months from 24 October 2023 to 10 April 2024 after obtaining permission from the institutional ethical committee (approval number: ASCOMS/IEC/2023Meeting2/FM/10). A total of 200 MBBS students were enrolled in the study after obtaining informed consent.

The following subjects were included in the study: MBBS students, both male and female, who were in the 18-25 age group and were willing to participate.

All the students were briefed about the nature of the study. A self-structured questionnaire was prepared after thorough literature reviews and was used to collect the data (Appendix A). The questions were designed keeping in view the objectives of the study and were made in such a way that they could be easily understandable to the participants. The data regarding variables like age, gender, educational status, semester, etc. were collected in a predesigned pro forma. The collected data was recorded in a Microsoft Excel sheet (Microsoft Corp., Redmond, WA) and analyzed with the help of IBM SPSS Statistics software for Windows, version 21.0 (IBM Corp., Armonk, NY).

## Results

A total of 200 students participated in the study, and their SM practice was analyzed. In the study, it was observed that there were 120 (60%) males and 80 (40%) females. The maximum subjects were in the age group of 21-23 years (120 students, 60%). The mean age was 21.4 ± 2.2 years. (mean ± SD) Out of the total subjects, 160 (80%) lived in urban areas and 40 (20%) lived in rural areas. Further, 170 (85%) students did not report any medical illness or medical history (Table 1).

**Table 1:** Demographic characteristics of the students (N = 200) The data have been represented as frequency (N) and percentage (%).

Demographic variable	Group	Frequency (n)	Percentage (%)
Gender	Male	120	60%
	Female	80	40%
Age	18-20	20	10%
	21-23	120	60%
	24-27	60	30%
Area of living	Urban	160	80%
	Rural	40	20%
Medical history/ illness	Yes	30	15%
	No	170	85%

Table 2 depicts that 130 (65%) students practiced SM for different ailments, whereas 70 (35%) students didn't. Eighty-three (64%) females were practicing SM, whereas only 47 males (36%) practiced the same in our study. Year-wise SM practices are also included, which showed that final-year students and interns practiced it the most. 

**Table 2:** Practice of self-medication among the participants The data have been represented as frequency (N) and percentage (%). In year-wise distribution, the total number of subjects is mentioned as frequency and percentage in the next column. The number of self-medicators is included in the brackets.

Demographic variable	Response	Frequency (n)	Percentage (%)
Practice of self-medication (SM)	Yes	130	65%
	No	70	35%
Gender-wise distribution	Females	83	64%
	Males	47	36%
Year-wise distribution	First-year	Total: 45 (SM practice: 21)	Total: 22.5% (SM:16.1%)
	Second year	Total: 43 (SM practice:20)	Total: 21.5% (SM:15.3%)
	Pre-final year	Total: 30 (SM practice:18)	Total: 15% (SM:13.8%)
	Final year	Total: 40 (SM practice:34)	Total: 25% (SM:26.1%)
	Intern	Total: 42 (SM practice:37)	Total: 21% (SM:28.4%)

**Figure 1:**
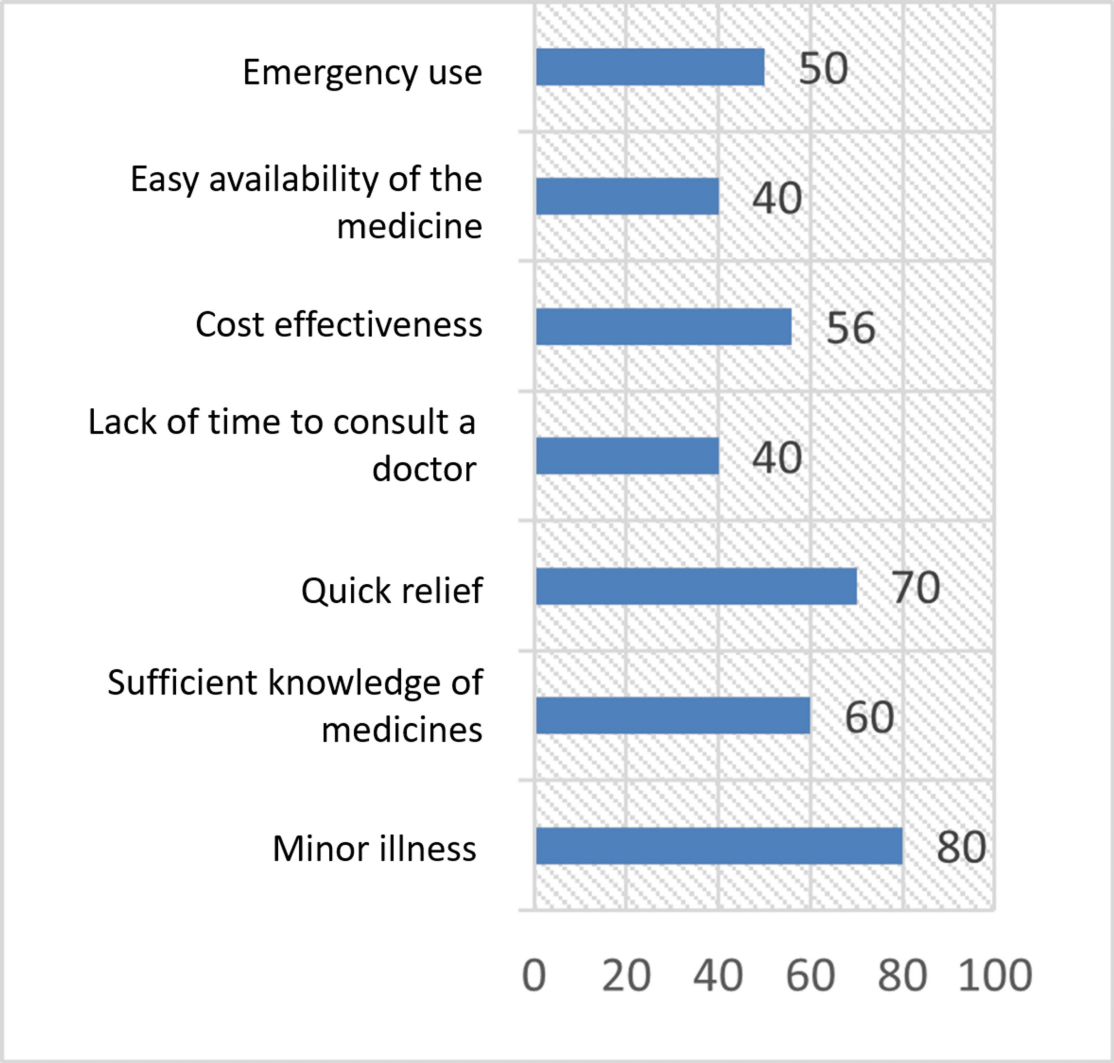
Reasons for self-medication The data have been represented as frequency (N).

The main reasons for choosing to self-medicate were minor illness (80 students, 40%), followed by quick relief (70 students, 35%), sufficient knowledge of medicines (60 students, 30%), cost-effectiveness (56 students, 28%), emergency use (50 students, 25%), and lack of time to consult a doctor, and easy availability of medicines (40 students each, 20%) (Table 3, Figure 1). 

**Table 3:** Reasons for self-medicating The data have been represented as frequency (N) and percentage (%).

Reason	Frequency (n)	Percentage (%)
Minor illness	80	40%
Sufficient knowledge of medicines	60	30%
Quick relief	70	35%
Lack of time to consult a doctor	40	20%
Cost-effectiveness	56	28%
Easy availability of medicine	40	20%
Emergency use	50	25%

Table 4 depicts the awareness of self-medication among the study participants. It was found that 152 (76%) students were aware of dose and frequency, 144 (72%) students were aware of adverse drug reactions, and 184 (92%) students checked the expiration date before using the medicines.

**Table 4:** Awareness of medications The data have been represented as frequency (N) and percentage (%).

Awareness	Response	Frequency	Number (%)
Aware of dose and frequency	Yes	152	76%
	No	48	24%
Awareness of adverse drug reactions	Yes	144	72%
	No	56	28%
Expiration date check	Yes	184	92%
	No	16	8%

**Figure 2:**
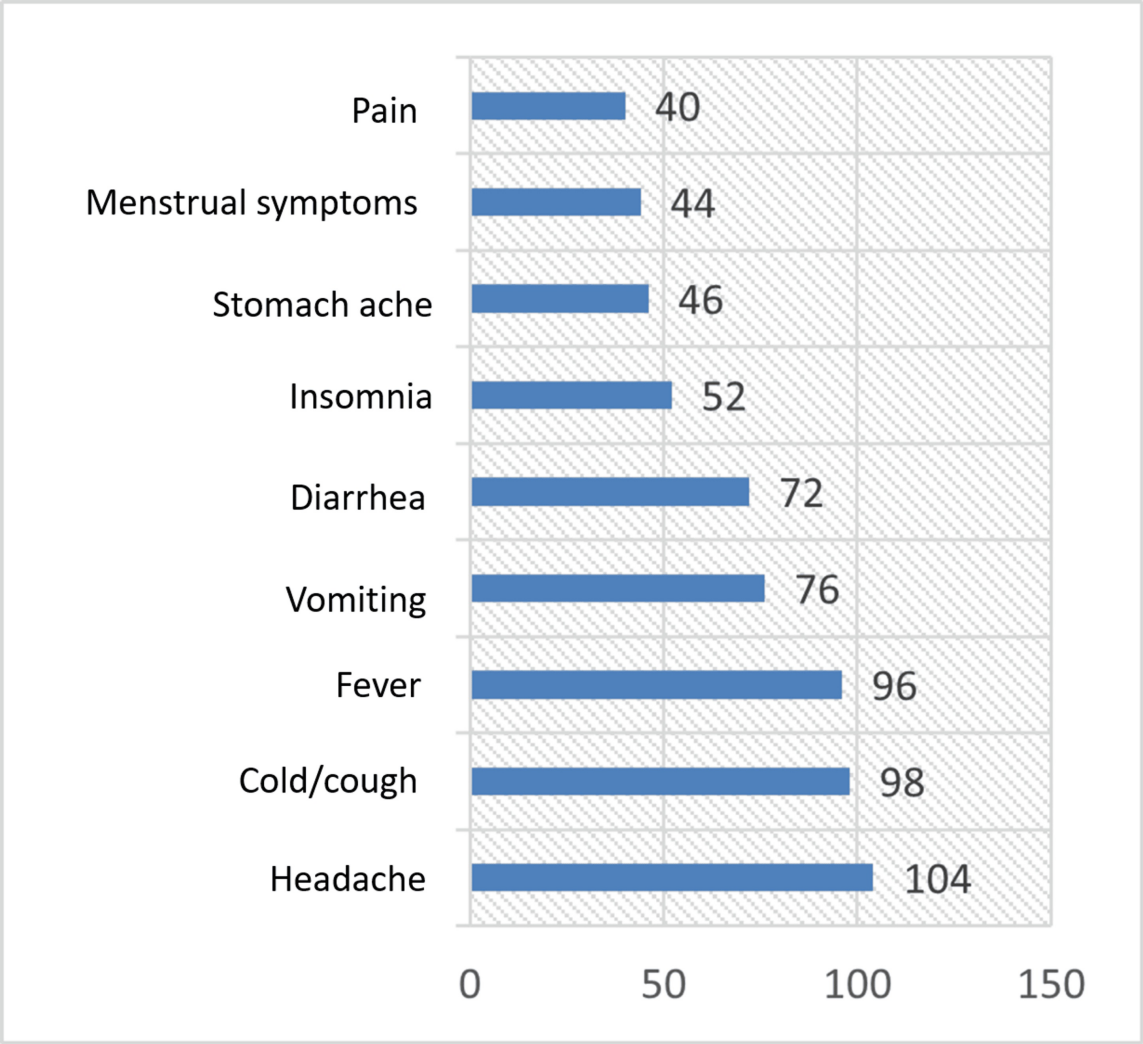
Common indications for self-medication The data have been represented as frequency (N).

Table 5 and Figure 2 depict the indications for self-medication. It was observed that common indications for self-medicating included headache (104 students, 52%), cough/ cold (98 students, 49%), fever (96 students, 48%), vomiting (76 students, 38%), diarrhea (72 students, 36%), insomnia (52 students, 26%), stomach ache (46 students, 23%), menstrual symptoms (44 students, 22%), and pain (40 students, 20%).

**Table 5:** Indications for self-medication The data have been represented as frequency (N) and percentage (%).

Indication	Number (N)	Percentage (%)
Headache	104	52%
Cough/ cold	98	49%
fever	96	48%
Vomiting	76	38%
Diarrhea	72	36%
Insomnia	52	26%
Stomach ache	46	23%
Menstrual symptoms	44	22%
Pain	40	20%

**Figure 3:**
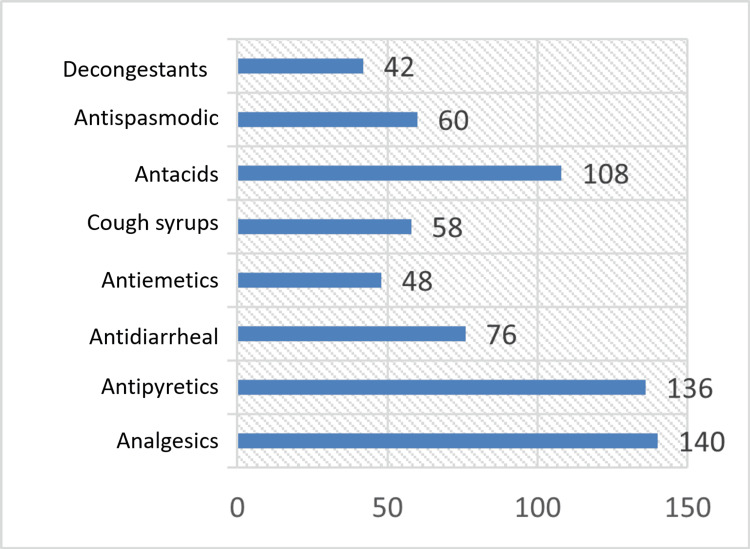
Commonly used drugs for self-medication The data have been represented as frequency (N).

Table 6 and Figure 3 depict the various types of medicines that were used without prescription, which included analgesics (140 students, 70%), antipyretics (136 students, 68%), antacids (108 students, 54%), antidiarrheal (76 students, 38%), antispasmodics (60 students, 30%), cough syrups (58 students, 29%), antiemetics (48 students, 24%), and decongestants (42 students, 21%). 

**Table 6:** Most commonly used drugs for self-medication The data have been represented as frequency(N) and percentage(%).

Drugs	Number (N)	Percentage (%)
Analgesics	140	70%
Antipyretics	136	68%
Antidiarrheal	76	38%
Antiemetics	48	24%
Cough syrups	58	29%
Antacids	108	54%
Antispasmodic	60	30%
Decongestant	42	21%

## Discussion

The least developed nations have seen the highest rates of irrational medication usage, particularly among young people and college students. Consequently, one important way to assess how students will behave when it comes to future prescription patterns for medications is to look into their perceptions of SM behaviors.

A total of 200 students participated in the study, and their SM practice was analyzed. In the study, it was observed that there were 120 (60%) males and 80 (40%) females. The maximum subjects were in the age group of 21-23 years, i.e., 120 (60%). The mean age was 21.4 ± 2.2 years. These observations are in accordance with the findings in a similar study conducted by Sankdia et al., where female students were more interested in taking SM compared to male students [6]. This may be because female students are more hesitant to go to the hospital or outpatient departments (OPDs) for minor illnesses. In another study conducted by Patil et al., there were more male students who practiced SM as compared to female students [7]. 

In our study, 130 (65%) students practiced SM for different ailments, whereas 70 (35%) students didn’t practice SM. The results are in accordance with the study conducted by Kumar et al. and Aljaouni et al., wherein the prevalence of SM among students was 78% and 64.8%, respectively [8,9].

It was found in our study that the main reasons for choosing to self-medicate were minor illness (80 students, 40%), followed by quick relief (70 students, 35%), sufficient knowledge of medicines (60 students, 30%), cost-effectiveness (56 students, 28%), emergency use (50 students, 25%), lack of time to consult a doctor, and easy availability of medicines (40 students each, 20%). Further, it was found that 152 (76%) students were aware of dose and frequency, 144 (72%) students were aware of adverse drug reactions, and 184 (92%) students checked the expiration date before using the medicines. Similar observations were reported in the studies conducted by Banerjee et al. and Badiger et al. [10,11]. In a study conducted by Kayalvizhi et al., most students practiced SM as it was time-saving, whereas, in a similar study conducted by Gupta et al., the most common reason for SM was for quick relief [12,13].

It was observed in our study that the common indications for self-medicating included headache (104 students, 52%), cough/cold (98 students, 49%), fever (96 students, 48%), vomiting (76 students, 38%), diarrhea (72 students, 36%), insomnia (52 students, 26%), stomach ache (46 students, 23%), menstrual symptoms (44 students, 22%), and pain (40 students, 20%). Melatonin in the form of gummies was the most commonly used drug for insomnia. The results of the studies conducted by Kayalvizhi et al. and Banerjee et al. showed that fever, headache, cough, and cold were the most common ailments for self-medication [10,12].

It was observed in our study that the various types of medicines that were used without prescription were analgesics (140 students, 70%), antipyretics (136 students, 68%), antacids (108 students, 54%), antidiarrheal (76 students, 38%), antispasmodics (60 students, 30%), cough syrups (58 students, 29%), antiemetics (48 students, 24%), and decongestants (42 students, 21%). Similar results were observed in the studies conducted by Badiger et al., Abay et al., and Zafar et al. [11,14,15]

A limitation of our study arose from the circumstance where each of the four professional years of MBBS in our institute consists of approximately 100 students each, and due to factors such as absenteeism, incomplete form submissions, or students declining to participate in the study for varied reasons, our sample size for the research was smaller than initially anticipated.

## Conclusions

The present study found a high prevalence of SM practice among medical students, especially among females. However, the knowledge and perception regarding SM were not insufficient. Self-medication may lead to medication misuse; therefore, it is recommended that better awareness be adopted among medical students about the necessity of consulting a physician before taking any medicine. Pharmacists should be made more aware of the situation and what effects it could have on the health of our society in general. 

## Appendices

Appendix A

Self-Structured Questionnaire Used in the Study

**Table 7:** Self-structured questionnaire to assess the participants' knowledge and perception regarding self-medication

Name:	
Age:	
Gender:	
Area of living:	
Any medical history/illness	
Do you practice self-medication for different ailments	Yes / No
What are the reasons for doing self-medication	Minor illness, sufficient knowledge of medicines, quick relief, lack of time to consult a doctor, cost-effectiveness, easy availability of medicines, emergency use
Are you aware of the dosage and frequency of drugs?	Yes / No
Are you aware of the adverse drug reactions?	Yes / No
Do you check manufacturing and expiration dates before using any drug?	Yes/ No
What are the indications for self-medication?	
What are the most commonly used drugs for self-medication?	
